# Strong-LAMP: A LAMP Assay for *Strongyloides* spp. Detection in Stool and Urine Samples. Towards the Diagnosis of Human Strongyloidiasis Starting from a Rodent Model

**DOI:** 10.1371/journal.pntd.0004836

**Published:** 2016-07-14

**Authors:** Pedro Fernández-Soto, Alicia Sánchez-Hernández, Javier Gandasegui, Cristina Bajo Santos, Julio López-Abán, José María Saugar, Esperanza Rodríguez, Belén Vicente, Antonio Muro

**Affiliations:** 1 Unidad de Investigación Enfermedades Infecciosas y Tropicales (e-INTRO), Instituto de Investigación Biomédica de Salamanca-Centro de Investigación de Enfermedades Tropicales de la Universidad de Salamanca (IBSAL-CIETUS), Facultad de Farmacia, Universidad de Salamanca, Salamanca, Spain; 2 Servicio de Parasitología, Centro Nacional de Microbiología, Instituto de Salud Carlos III, Madrid, Spain; QIMR Berghofer Medical Research Institute, AUSTRALIA

## Abstract

**Background:**

*Strongyloides stercoralis*, the chief causative agent of human strongyloidiasis, is a nematode globally distributed but mainly endemic in tropical and subtropical regions. Chronic infection is often clinically asymptomatic but it can result in severe hyperinfection syndrome or disseminated strongyloidiasis in immunocompromised patients. There is a great diversity of techniques used in diagnosing the disease, but definitive diagnosis is accomplished by parasitological examination of stool samples for morphological identification of parasite. Until now, no molecular method has been tested in urine samples as an alternative to stool samples for diagnosing strongyloidiasis. This study aimed to evaluate the use of a new molecular LAMP assay in a well-established Wistar rat experimental infection model using both stool and, for the first time, urine samples. The LAMP assay was also clinically evaluated in patients´ stool samples.

**Methodology/Principal Findings:**

Stool and urine samples were obtained daily during a 28-day period from rats infected subcutaneously with different infective third-stage larvae doses of *S*. *venezuelensis*. The dynamics of parasite infection was determined by daily counting the number of eggs per gram of feces from day 1 to 28 post-infection. A set of primers for LAMP assay based on a DNA partial sequence in the 18S rRNA gene from *S*. *venezuelensis* was designed. The set up LAMP assay (namely, Strong-LAMP) allowed the sensitive detection of *S*. *venezuelensis* DNA in both stool and urine samples obtained from each infection group of rats and was also effective in *S*. *stercoralis* DNA amplification in patients´ stool samples with previously confirmed strongyloidiasis by parasitological and real-time PCR tests.

**Conclusions/Significance:**

Our Strong-LAMP assay is an useful molecular tool in research of a strongyloidiasis experimental infection model in both stool and urine samples. After further validation, the Strong-LAMP could also be potentially applied for effective diagnosis of strongyloidiasis in a clinical setting.

## Author Summary

Human strongyloidiasis, a soil-transmitted infection mainly caused by *Strongyloides stercoralis*, is one of the most neglected among the so-called neglected tropical diseases (NTDs). The difficult diagnosis lead to an underreporting of infection rates. Strongyloidiasis can easily be misdiagnosed because many infections remain asymptomatic and the lack of sensitivity of the conventional fecal-based techniques for morphologically identification of infective larvae in feces. Although serologic tests are useful, a limitation in standardization to avoid cross-reactions still remains. There is an urgent need to improve more sensitive and specific diagnostic tests, particularly in immunocompromised patients or candidates to immunosuppressive treatments. Several molecular approaches for *Strongyloides* spp. DNA detection have already been assayed, but they have a very limited use in routine diagnostic, particularly in endemic areas. In addition, all molecular approaches for *Strongyloides* spp. DNA detection have always been mainly assayed for stool samples and no other more advantageous biological samples, such as urine, have been investigated for molecular purposes. In this study we have developed, for the first time, a molecular assay using LAMP methodology as a simple, sensible and robust method for the detection of *S*. *venezuelensis* DNA in a well-established Wistar rats experimental infection in both stool and urine samples. The LAMP assay was also successfully evaluated in patients´ stool samples. Our LAMP assay (Strong-LAMP) is an useful molecular tool in a strongyloidiasis experimental infection model and could be a potential field-friendly diagnostic test in a clinical setting, following further validation.

## Introduction

Strongyloidiasis, a soil-transmitted helminth human infection, is considered by World Health Organization (WHO) as a neglected condition affecting an estimated 30–100 million people worldwide [1]. The accuracy of these estimates remains actually uncertain due to lack of efficient guidelines for screening the population in epidemiological surveys [2, 3]. At least, two species of nematodes of the genus *Strongyloides*, namely *Strongyloides stercoralis* (the most common human pathogen species) and *S*. *fuelleborni*, are known to infect humans causing strongyloidiasis [4, 5, 6]. Human infection is primarily acquired by the filariform larvae (the infective third-stage larvae, iL3) penetrating the skin or mucous membranes through unprotected contact with contaminated soil [7]. *S*. *stercoralis* biology is complex involving two separate life cycles, the free-living heterogonic cycle and a parasitic cycle [8, 9]. The exceptional ability of this parasite to replicate in the human host permits ongoing cycles of autoinfection thus resulting in a chronic strongyloidiasis that can therefore persist for several decades without further exposure to a new exogenous infection [6]. Inmunocompetent patients with uncomplicated strongyloidiasis usually develop an asymptomatic, mildly symptomatic or chronic infection, which are typically associated with low intestinal worm burdens and intermittent larval excretion [10, 2]. However, a deregulation of the host´s immune response during the latent infection may lead in an uncontrolled multiplication of the parasites (hyperinfection syndrome) which can be life-threatening [6, 2, 10, 11, 7] with mortality rates of up 87% [12, 13]. Thus, detecting latent cases of *S*. *stercoralis* is crucial to decrease morbidity and mortality of the infection.

The diagnosis of strongyloidiasis is suspected when clinical signs and symptoms, eosinophilia or serologic findings are observed [14, 6], but definitive diagnosis is accomplished by parasitological examination of stool samples allowing the morphological identification of *S*. *stercoralis*, including direct smear in saline, the spontaneous sedimentation method [15], centrifugation [16], the Baermann´s technique [17], the agar plate culture [18] and the Harada-Mori´s filter paper culture method [19]. These methods have classically low sensitivity because of the low and irregular load of larvae in the feces [6]; the collection of a larger number of stool samples on alternate days instead of a single one and the combination of several diagnostic methods may increase the sensitivity [20]. On the other hand, several immunological methods have been described for diagnosing strongyloidiasis showing a high sensitivity when compared with parasitological methods but a limitation in the standardization of more specific serological tests in order to avoid the possibility of cross-reaction with other helminths still remains [21, 22]. Several DNA-based techniques (i.e. single-PCR, nested-PCR, PCR-RFLP, real-time PCR) have provided useful alternatives not only for identification of *Strongyloides* species [23, 24] but also for *S*. *stercoralis* DNA detection in feces with high accuracy in the diagnosis of strongyloidiasis [22, 25, 26, 27]. Nevertheless, such molecular methods have a very limited use in routine diagnostic, particularly under field conditions in endemic areas requiring special equipment manipulated by trained personnel. Thus, the development of new, simple, applicable and cost-effective alternative molecular assays is necessary to diagnose human strongyloidiasis, mainly in those immunocompromised individuals in which the infection can be fatal.

At present, there is a nucleic acid amplification method named loop-mediated isothermal amplification (LAMP) [28]. Compared to PCR, the simplicity of the LAMP method makes it suitable for field testing in developing countries [29, 30], and many LAMP reactions have already been developed for molecular detection and diagnostics of infectious diseases, including parasitic diseases [31]. In this sense, a first LAMP assay for the detection of *S*. *stercoralis* in feces has been recently developed and preliminary evaluated with human stool samples [32]. To date, all new successfully approaches for molecular methods to be used for *Strongyloides* spp. DNA detection have been focused in analyzing mainly stool samples and no other type of sample, such as urine, has been considered for the detection of parasite DNA. Urine is a biological sample that would have a number of advantages in diagnosis of strongyloidiasis over the stool samples since it has less inconvenience to obtain from patients as well as it is easier in handling and storing. It has been demonstrated that small amounts of cell-free circulating DNA are able to pass the kidney barrier and end up in urine [33, 34, 35]; this circulating DNA from the bloodstream that passes into the urine can be isolated and used in diagnostic applications.

This study aimed to assess the diagnostic utility of a new designed LAMP assay in an active experimental rodent strongyloidiasis in parallel with parasitological method by direct fecal examination. We used as a source for *Strongyloides* spp. DNA detection both stool and, for the first time, urine samples from rats experimentally infected with different doses of *S*. *venezuelensis* iL3. The LAMP developed in this work (namely, Strong-LAMP) was shown to be sensitive and specific in detecting *Strongyloides* spp. DNA. The potential diagnostic applicability of the Strong-LAMP could be also demonstrated on a number of human clinical stool samples with previously parasitological and real-time PCR confirmed strongiloidiasis.

## Methods

### Ethics statement

The study protocol was approved by the institutional research commission of the University of Salamanca. Ethical approval was obtained from the Ethics Committee of the University of Salamanca (protocol approval number 48531), which approved the animal protocol. Animal procedures in this study complied with the Spanish (Real Decreto RD53/2013) and the European Union (European Directive 2010/63/EU) guidelines on animal experimentation for the protection and humane use of laboratory animals and were conducted at the accredited Animal Experimentation Facility of the University of Salamanca (Register number: PAE/SA/001). The human stool samples used in this study were obtained as part of public health diagnostic activities at Severo Ochoa and Gregorio Marañón Hospitals, Madrid, Spain. A standardized epidemiological questionnaire and clinical information were obtained from each participant included in the study. Participants were given detailed explanations about the aims, procedures and possible benefit of the study. Written informed consent was obtained from all subjects and samples were coded and treated anonymously. The study received the approval of the Committee of Research Ethics and Animal Welfare from the Instituto de Salud Carlos III (PI number: CEI PI06_2012-v2).

### Animals

Twelve six-week-old male Wistar rats weighing 150–175 g (Charles River Laboratories, Barcelona, Spain) were used in our study as the source for stool and urine samples. Animals were housed at the accredited Animal Experimentation Facility of the University of Salamanca in individual metabolic polycarbonate cages and placed in humidity and temperature controlled environment with a 12 hour photoperiod and received sterilized food and water *ad libitum*. Animals were monitored regularly by qualified members in animal welfare at the Animal Experimentation Facility of the University of Salamanca.

### *Strongyloides venezuelensis* experimental infection

*Strongyloides venezuelensis* used in this study was obtained from a strain originally used in the Department of Parasitology, University of Minas Gerais, Belo Horizonte, Brazil. This strain has been maintained by serial passages in laboratory rats routinely infected in the Laboratory of Parasitic and Molecular Immunology, CIETUS, University of Salamanca. Feces from infected rats were cultured using vermiculite mixed with distilled water at 28°C for 3–7 days and infective third-stage larvae (iL3) which came out of the feces were then collected and concentrated by using the Baermann extraction method as described elsewhere [36]. Recovered larvae were washed in phosphate-buffered saline (PBS) and their viability was checked using a light microscope prior to infection. The number of viable iL3 was determined and the animals were afterwards infected subcutaneously with different iL3 doses of *S*. *venezuelensis* to ensure a potential range of low, middle and high fecal egg production during the development of infection [37], as follows: group one (n = 3; each rat infected with 40 iL3), group 2 (n = 3; each rat infected with 400 iL3), group 3 (n = 3; each rat infected with 4,000 iL3) and group 4 (n = 3; non infected, as control group). During the 28-day infection, the animals were housed individually in metabolic cages, thus allowing separate collection of urine and feces from rodents and also to eliminate the possibility of rats re-infecting themselves from fecal sources during the experimental period. Infected rats were euthanatized in a CO_2_ gas chamber 29 days after the infection.

### Rat samples and monitoring *S*. *venezuelensis* infection

#### Rat stool samples

Feces were taken daily from all rats housed individually in the metabolic cages throughout the 28-day infection. The three stool samples taken daily from each group of three rats (infected and non-infected) were pooled and the resulting mix was treated as a single sample that was subsequently divided into two portions: one (0.7–1 g, approximately) was freeze-dried stored at -20°C to be used afterward for DNA extraction and another was kept at ambient temperature by adding 3% formalin to the remaining feces (3–5 mL, depending on the volume of fecal sample) to preserve parasite eggs for later counting.

The dynamics of *S*. *venezuelensis* infection was determined by daily counting the number of eggs per gram of feces (EPG) by a modified McMaster technique [38] from day 1 post-infection (p.i.) until day 28 p.i.. The EPG counting was performed by triplicate in the daily pool of stool samples preserved in formalin obtained from each infected group of animals as mentioned above. Results obtained were expressed as mean±SE.

#### Rat urine samples

Urine samples were also individually taken daily from all animals throughout the 28-day infection. The three urine samples taken daily from each group of three rats (infected and non-infected) were also pooled and the resulting mix was treated as a single sample to minimize the number of subsequent reactions. Each daily pool was afterward divided into two aliquots: one aliquot (1 mL) was immediately used for DNA extraction for molecular analyses and another (a variable remaining volume) was stored at -20°C.

### Human stool samples and parasitological tests

Human stool samples (n = 12) were obtained from outpatients (including Spanish nationals, immigrants, tourists and aid workers) attending Severo Ochoa and Gregorio Marañón Hospitals in Madrid, Spain, during June 2010 to June 2012 as a part of a collaborative research study on human strongyloidiasis. Those patients showed significant levels of IgE, eosinophilia or other symptoms suggestive of disease. Stool samples were examined after arrival by qualified laboratory technicians. Eleven of these 12 stool samples (nos. 030, 140, 231, 232, 338, 339, 069, 259, 331, 468 and 126) were subjected to different parasitological methods as screening tests for strongyloidiasis, including microscopic examination (MOE) for the presence of rabditiform larvae in direct fecal smears, agar plate culture (APC) or Harada-Mori´s filter paper culture method (HMM). Unfortunately, for one sample (no. 496) was not possible to perform any of the parasitological methods. Strongyloidiasis was confirmed in 7/11 stool samples by one or more parasitological tests applied. Four samples (nos. 259, 331, 468 and 126) were found to be negative; however, in two of these negative samples (nos. 468 and 126) eggs from *Taenia saginata* and "hookworm", respectively, could be observed upon microscopic inspection. All patients´ samples were obtained before treatment with ivermectin. Thereafter, patients´ stool samples were sent to the Instituto de Salud Carlos III (ISCIII), Madrid, Spain, for further DNA extraction and molecular analyses by real-time PCR (RT-PCR) as described below. Table 1 shows the patients´ stool sample numbers, the parasitological tests applied at Hospitals as well as results obtained in parasitological and molecular tests performed.

**Table 1:** Patients´ stool sample numbers included in this study and parasitological and molecular tests applied for *Strongyloides stercoralis* detection.

	Patients´ stool samples											
	030	140	231	232	338	339	496^a^	069	259	331	468^b^	126^c^
Tests												
APC	+	+			+	+		+	-	-	-	-
HMM	+	+							-	-	-	-
MOE		+	+	+	+	+			-	-	-	-
RT-PCR	+	+	+	+	+	+	+	-	-	-	-	-
LAMP	+	+	+	+	+	+	+	+	-	-	-	-

APC, agar plate culture; HMM, Harada-Mori´s method; MOE, microscopic examination

´+´ and ´-´ represent positive and negative results, respectively.

^a^no parasitological method applied.

^b^positive for *Taenia saginata*.

^c^positive for hookworm.

### DNA extraction for molecular analyses

#### Parasites DNA samples

*S*. *venezuelensis* genomic DNA was extracted from iL3 using the Dneasy Blood & Tissue kit (QIAGEN, Hilden, Germany) following the manufacturers´ instructions. DNA was eluted with 100 μL AE buffer and the concentration of *S*. *venezuelensis* DNA iL3 was measured three times by spectrophotometry using a Nanodrop ND-100 spectrophotometer (Nanodrop Technologies) to obtain an average concentration and then diluted with ultrapure water to a final concentration of 5 ng/μL. Subsequently, serial 10-fold dilutions from *S*. *venezuelensis* DNA iL3 thus obtained were also prepared with ultrapure water ranging from 1x10^-1^ (0.5 ng/μL) to 1x10^-10^ (0.0005 fg/μL) and stored at -20°C until use. DNA thus prepared was used as a positive control in all PCR and LAMP reactions as well as for assessing sensitivity of both assays.

Additionally, to determine the specificity of both PCR and LAMP assays to amplify only *S*. *venezuelensis* DNA, a total of 22 DNA samples from several helminths and protozoa were used as heterogeneous control samples, including *Schistosoma mansoni*, *S*. *intercalatum*, *S*. *haematobium*, *S*. *japonicum*, *S*. *bovis*, *Fasciola hepatica*, *Loa loa*, *Brugia pahangi*, *Dicrocoelium dendriticum*, *Calicophoron daubneyi*, *Hymenolepis diminuta*, *Taenia taeniformis*, *Anisakis simplex*, *Trichinella spiralis*, *Echinostoma caproni*, *Echinococcus granulosus*, *Cryptosporidium parvum*, *Giardia intestinalis*, *Entamoeba histolytica*, *Plasmodium malariae*, *P*. *ovale* and *P*. *vivax*. Concentration of all DNA samples was measured by the same method as described for *S*. *venezuelensis* iL3 DNA and then also diluted with ultrapure water to a final concentration of 0.5 ng/μL. In order to look for protein contaminations a common purity check by measuring the A_260_/A_280_ ratio was made for all samples. All these DNA samples were kept at -20°C until use in molecular assays.

#### DNA from rat stool samples

Approximately 200 mg of frozen stool samples from each daily pool obtained from each group of animals were used for DNA extraction using the NucleoSpin Tissue Kit (Macherey-Nagel, GmbH & Co., Germany)- according to the modified protocol for DNA extraction from stool- following the manufacturers´ instructions. All DNA samples obtained from feces from non-infected rats (group 4) were pooled and the resulting mix was treated as a single negative control sample to minimize the number of subsequent molecular reactions. Purified DNA samples were stored at -20°C until use.

#### DNA from rat urine samples

The DNA of 1mL of daily pooled urine samples from each group of rats was extracted using the i-genomic Urine DNA Extraction Mini Kit (Intron Biotechnology) following the manufacturers´ instructions. Once extracted, the purified DNA of urine samples was stored at -20°C until use in molecular assays.

#### DNA from patients´ stool samples

Prior to DNA extraction, 1 g of each stool sample was resuspended in 8 mL of saline solution and concentrated using Bioparapred-Midi columns (Leti Diagnostics, Barcelona, Spain). Then, the supernatants were discarded and 200 mg of the pellets obtained were used for total DNA extraction using the QIAmp DNA Stool Mini kit (QIAGEN, Hilden, Germany) following the manufacturer´s instructions. Purified DNA samples were stored at -20°C until use in RT-PCR at ISCIII, Madrid, Spain, and afterwards in PCR and LAMP assays at CIETUS, Salamanca, Spain.

### Real-time PCR amplification

Patients´ stool samples included in this study were firstly tested by a RT-PCR optimized at ISCIII, Madrid, Spain, as described by Saugar et al. [27]. Briefly, the RT-PCR was standardized in laboratory settings using the specific primers Stro18S-1530F (5′-GAATTCCAAGTAAACGTAAGTCATTAGC-3′) and Stro18S-1630R (5′-TGCCTCTGGATATTGCTCAGTTC-3′) to amplified a 101 base pair (bp) region of *S*. *stercoralis* 18S rRNA (Gene Bank accession no. AF279916.2) as previously described by Verweij et al. [25]. The amplification was performed with a 25 μL reaction mix containing 5 μL of DNA extracted from stool samples, 1X Quantimix Easy Master Mix (Biotools B&M Laboratories), 0.2 μM of each Stro18S-1530F and Stro18S-1630R primer and 0.5 μL of 50X SYBR Green I (Invitrogen). The program consisted of an initial step of 15 min at 95°C followed by 50 cycles of 10 s at 95°C, 10 s at 60°C and 30 s at 72°C. The reaction and fluorescence detection were performed on the Corbett Rotor-Gene 6000 real-time PCR System (QIAGEN, Hilden, Germany) and The Rotor Gene 6000 Series software v.1.7 was used for data analysis. In each RT-PCR run both negative (DNA from uninfected stool sample) and positive (DNA from stool samples artificially infected with different amounts of *S*. *venezuelensis* iL3 DNA) controls were routinely included.

### LAMP primers design

**Fig 1:**
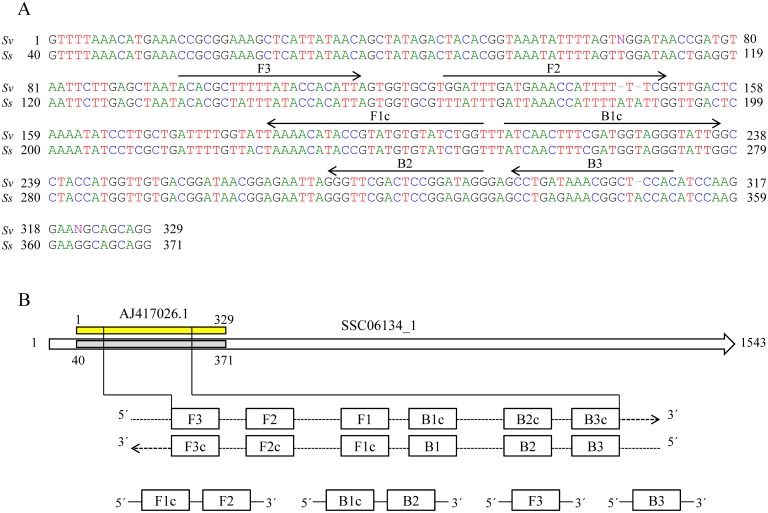
Design of LAMP primers for detection of *Strongyloides venezuelensis*. (A) Nucleotide sequence alignment of the 329 bp selected sequence (AJ417026.1) for *S*. *venezuelensis* (Sv) and a partial sequence in contig SSC06134_1 for *S*. *stercoralis* (Ss). The sequences used for LAMP primers are indicated by arrows. (B) Schematic representation of AJ417026.1 and SSC06134_1 sequences and the primers used in this study. Construction of the inner primers FIP (F1c+F2) and BIP (B1c+B2) are shown. F1c and B1c are complementary to F1 and B1, respectively.

After searching on literature reports to identify potential sequences of DNA to be used in detection of *Strongyloides* spp., a 329 nucleotide bp corresponding to a linear genomic DNA partial sequence in the 18S rRNA gene from *S*. *venezuelensis* was selected and retrieved from GenBank (Accession No. AJ417026.1) for the design of specific primers [39]. A BLASTN search and alignment analysis [40] indicated that the sequence had 94–99% similarity with other sequences reported for *Strongyloides* spp. and no regions of similarity between this sequence and other sequences reported for possible human pathogens were detected. The 329 bp sequence selected was also tested *in silico* for similarity in the currently available genome databases for *S*. *stercoralis* at NemBase4 (www.nematodes.org) and a 94% identity with a partial sequence in contig SSC06134_1 annotated for the parasite was obtained. Forward and backward outer primers (F3 and B3) and forward and backward inner primers (FIP: F1c-F2 and BIP: B1c-B2, respectively) were designed using the online primer design utility, Primer Explorer v.4 (Eiken Chemical Co., Ltd., Japan; http://primerexplorer.jp/e/). Several LAMP primer sets were suggested by the software and further refinement in primer design was developed manually based on the criteria described in “A Guide to LAMP primer designing” (http://primerexplorer.jp/e/v4_manual/index.html). LAMP primers sequences finally selected are indicated in Table 2 and their positions relative to the 329 bp target sequence of *S*. *venezuelensis* compared with the 94% similarly partial sequence of *S*. *stercoralis* in contig SSC06134_1 are shown in Fig 1. All the primers were of HPLC grade (Thermo Fisher Scientific Inc., Madrid, Spain); the lyophilized primers were resuspended in ultrapure water to a final concentration of 100 pmol/μL and stored at -20°C until use.

**Table 2:** Sequences of LAMP primers for the amplification of the linear genomic DNA partial sequence in the 18S rDNA from *Strongyloides venezuelensis* (GenBank Accession No. AJ417026.1).

Primer^a^	Length (bp)	Sequence (5´-3´)
F3	21	ACACGCTTTTTATACCACATT
B3	18	GTGGAGCCGTTTATCAGG
FIP (F1c+F2)	49	ACCAGATACACATACGGTATGTTTT-GGATTTGATGAAACCATTTTTTCG
BIP (B1c+B2)	43	ATCAACTTTCGATGGTAGGGTATTG-CCTATCCGGAGTCGAACC

^*a*^ F3, forward outer primer; B3, reverse outer primer; FIP, forward inner primer (comprising F1c and F2 sequences); BIP, reverse inner primer (comprising B1c and B2 sequences).

### LAMP F3 and B3 primer specificity tested by PCR

The outer LAMP primer pair, designated F3 and B3 (Table 2), was firstly tested for *S*. *venezuelensis* specificity by a touchdown-PCR to verify whether the correct target was amplified. The PCR assay was conducted in 25 μL reaction mixture containing 2.5 μL of 10x buffer, 1.5 μL of 25 mmol/L MgCl_2_, 2.5 μL of 2.5 mmol/L dNTPs, 0.5 μL of 100 pmol/L F3 and B3, 2 U *Taq*-polymerase and 2 μL (10 ng) of DNA template. Initial denaturation was conducted at 94°C for 1 min, followed by a touchdown program for 15 cycles with successive annealing temperature decrements of 1.0°C (from 57°C to 52°C) every 2 cycles. The specificity of PCR was also tested with a panel of 22 heterogeneous DNA samples from other parasites included in the study. Besides, the sensitivity of the PCR was also assayed to establish the detection limit of *S*. *venezuelensis* DNA with 10-fold serial dilutions prepared as mentioned above.

### Optimization of LAMP assay

We tried to evaluate the LAMP primer set designed by using different *in house* reaction mixtures each containing a different *Bst* polymerase (namely, *Bst* DNA polymerase Large Fragment, *Bst* DNA polymerase 2.0 and *Bst* DNA polymerase 2.0 WarmStart; New England Biolabs, UK) as well as varying concentration of betaine (Sigma, USA) and supplementary MgSO_4_ (New England Biolabs, UK) to compare results in *S*. *venezuelensis* DNA amplification.

Thus, LAMP reactions mixtures (25 μL) contained 40 pmol of each FIP and BIP primers, 5 pmol of each F3 and B3 primers, 1.4 mM of each dNTP (Bioron), 1x ThermoPol Reaction Buffer -20 mM Tris-HCl (pH 8.8), 10 mM KCl, 10 mM (NH_4_)_2_SO_4_, 2 mM MgSO_4_, 0.1% Triton X-100; New England Biolabs, UK- (when using *Bst* Polymerase Large Fragment) or 1x Isothermal Amplification Buffer -20 mM Tris-HCl (pH 8.8), 50 mM KCl, 10 mM (NH_4_)_2_SO_4_, 2 mM MgSO_4_, 0.1% Tween20; New England Biolabs, UK- (when using either *Bst* DNA polymerase 2.0 or *Bst* DNA polymerase 2.0 WarmStart), betaine (ranging 0.8, 1, 1.2, 1.4 or 1.6 M), supplementary MgSO_4_ (ranging 2, 4, 6 or 8 mM) and 8 U of the tested *Bst* polymerase in each case with 2 μL of template DNA.

All LAMP reactions mixtures were performed in 0.5-mL micro centrifuge tubes that were incubated in a heating block (K Dry-Bath) at a range of temperatures (61, 63 and 65°C) for 60 min to optimize the reaction conditions and then heated at 80°C for 5–10 min to inactivate the enzyme and thus to terminate the reaction. In each case, the optimal temperature was determined and used in the subsequent tests. As the LAMP reaction is highly sensitive, possible DNA contamination and carry-over of amplified products were prevented by using sterile tools at all times, performing each step of the analysis in separate work areas, minimizing manipulation of the reaction tubes and even closing them with a plastic paraffin film. Template DNA was replaced by ultrapure water as negative control in each LAMP reaction.

### Detection of LAMP products

Amplified DNA in the LAMP reaction causes turbidity due to the accumulation of magnesium pyrophosphate, a by-product of the reaction. Once the reaction was finished and following a brief spin of the reaction tubes, the turbidity of reaction mixture was visually inspected by naked eyes. The LAMP amplification results could also be visually inspected by adding 2 μL of 1:10 diluted 10,000X concentration SYBR Green I (Invitrogen) to the reaction tubes. To avoid as much as possible the potential risk of cross-contamination with amplified products, all tubes were briefly centrifuged and carefully opened before adding the fluorescent dye. Green fluorescence was clearly observed in successful LAMP reaction, whereas it remained original orange in the negative reaction. The LAMP products (3–5 μL) were also monitored using 2% agarose gel electrophoresis stained with ethidium bromide, visualized under UV light and then photographed using an ultraviolet image system (Gel documentation system, UVItec, UK).

### Analytical specificity and limit of detection of the LAMP assay

The specificity of the LAMP assay to amplify only *S*. *venezuelensis* DNA was tested against 22 DNA samples obtained from other parasites used as controls as mentioned above. To determine the lower detection limit of the LAMP assay, genomic DNA from *S*. *venezuelensis* 10-fold serially diluted as mentioned above was subjected to amplification in comparison with the PCR using outer primers F3 and B3.

### Evaluation of the LAMP assay

To evaluate the ability of the LAMP assay designed to amplify *S*. *venezuelensis* DNA in real samples, we used DNA extracted from the pooled feces and urine samples taken daily from each experimentally infected group of rats with different iL3 doses. To check whether LAMP assay designed was also able to amplify DNA from *S*. *stercoralis* in clinical samples, we used the patients´ stool samples included in the study. In all amplification assays, positive (*S*. *venezuelensis* DNA) and negative (DNA mix from non-infected rats stool or urine samples or ultrapure water) controls were always included.

## Results

### Monitoring of *S*. *venezuelensis* infection by counting eggs in rat stool samples

**Fig 2:**
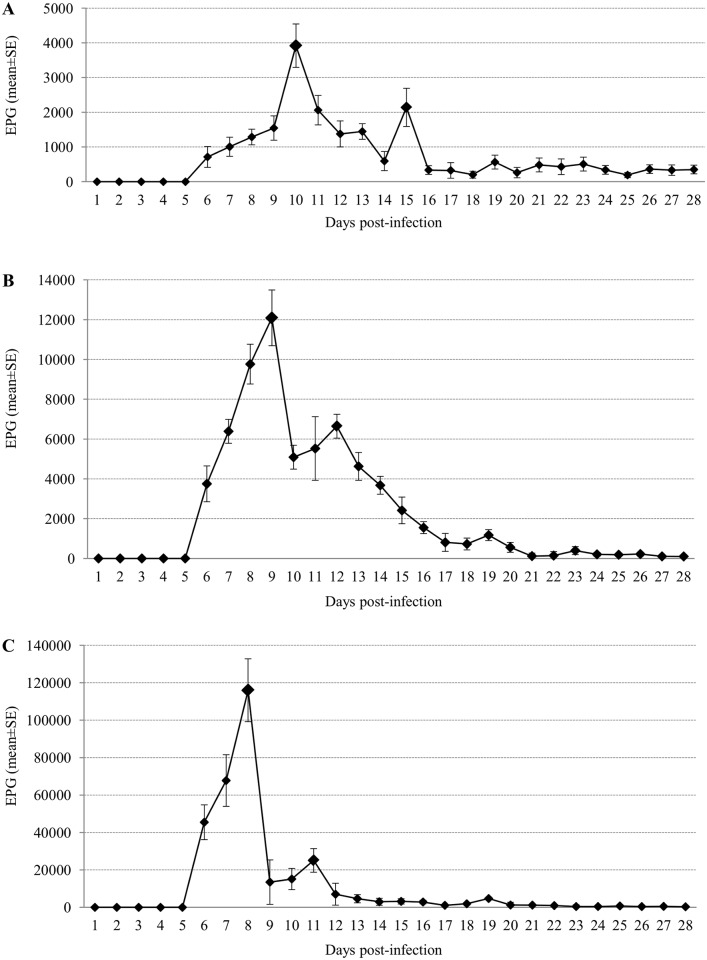
Monitoring of *Strongyloides venezuelensis* infection by counting eggs in rats’ stool samples. Rats were experimentally infected with different infective third-stage larvae (iL3) doses of *S*. *venezuelensis*. (A) Group 1: rats infected with 40 iL3. (B) Group 2: rats infected with 400 iL3. (C) Group 3: rats infected with 4,000 iL3. X axis represent days post-infection. Y axis represent number of eggs per gram of feces (EPG) and (mean±SE).

In the three infected groups, parasite eggs were detected for the first time in feces on the 6th day p.i. regardless of the initial infecting doses (Fig 2). The maximal fecal egg count was 3,921 EPG on day 10 p.i in group 1 (Fig 2A), 12,092 EPG on day 9 p.i. in group 2 (Fig 2B) and 116,016 EPG on day 8 p.i. in group 3 (Fig 2C).

### Analytical specificity and limit of detection of PCR using outer primers

When a PCR verification reaction was performed using primers F3 and B3 to amplify *S*. *venezuelensis* DNA a 215 bp PCR product was successful amplified; the minimum amount of DNA detectable by PCR was 0.01 ng. When a panel of 22 DNA samples from other parasites were subjected to this PCR assay, amplicons were never obtained (S1 Fig).

### Setting up LAMP assay

Considering the most consistent color change by adding SYBR Green I into the tubes, the intensity of the ladder-like pattern on agarose gel electrophoresis as well as reproducibility of tests, the best amplification results were always obtained when the reaction mixtures contained *Bst* DNA polymerase 2.0 or *Bst* DNA polymerase 2.0 WarmStart combined with 1 M of betaine and supplementary 6 mM of MgSO_4_ and the reaction tubes were incubated at 63°C for 60 min. We also obtained amplification results when using *Bst* polymerase LF in such conditions, but the color change in reaction tubes as well as the intensity of the ladder-like pattern on agarose were always less evident compared to that obtained when using the other two enzymes; additionally, we did not get a good reproducibility of amplification trials so we discarded to use *Bst* polymerase LF in the following applications. When we evaluated the sensitivity of both LAMP reaction mixtures containing *Bst* polymerase 2.0 and *Bst* polymerase 2.0 WarmStart, the limit of detection in *S*. *venezuelensis* DNA amplification was 0.1 ng and 0.01 ng, respectively. As sensitivity was tenfold higher when using *Bst* polymerase 2.0 WarmStart, the reaction mixture containing this enzyme was used in assessing the specificity of the LAMP assay. Then, the LAMP assay was positive only for *S*. *venezuelensis* and no positive DNA products were observed when other parasites species were used as templates (S2 Fig). Thereby, the LAMP reaction mixture containing *Bst* polymerase 2.0 WarmStart was set up as the most suitable to analyze all the samples included in the study and thenceforth was namely Strong-LAMP.

In addition, all non-template controls were negative for each batch of LAMP reactions, thus indicating that there was no cross contamination and that with the primers set used there was no template free amplification [41, 42].

### Examination of rat stool samples by LAMP

**Fig 3:**
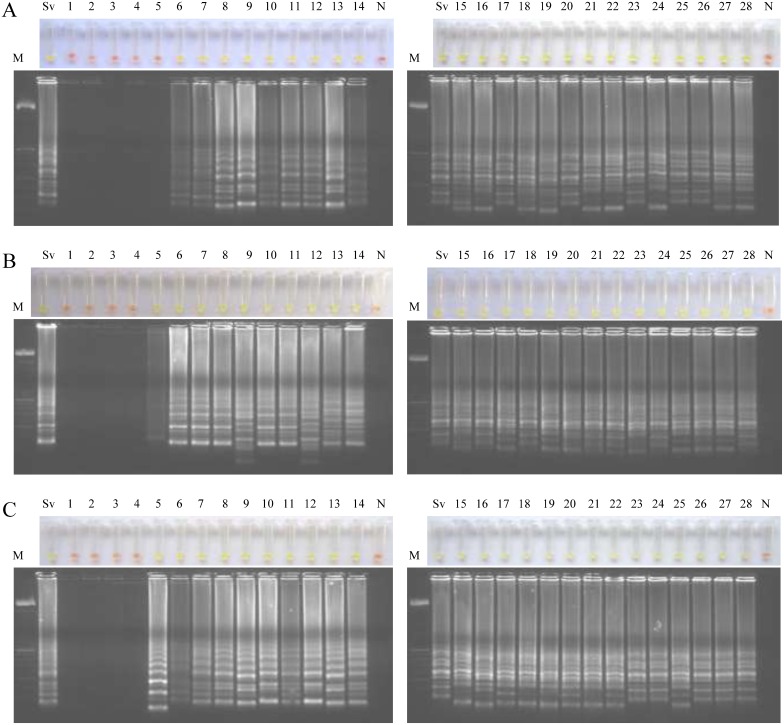
Examination of rats’ stool samples by Strong-LAMP assay. Analysis of each daily pool of stool samples obtained from each infection group of rats during a 28-day period. (A) Group 1, infected with 40 iL3. (B) Group 2, infected with 400 iL3. (C) Group 3, infected with 4,000 iL3. Lanes M, 50 bp DNA ladder (Molecular weight marker XIII, Roche); Lanes Sv, genomic DNA from *S*. *venezuelensis* (1 ng); lanes 1–28, pooled feces samples obtained at days 1–28 post-infection; lanes N, negative controls (pooled feces from non-infected rats, group 4).

We tested by LAMP each daily pool of stool samples obtained from each infection group of animals during a 28-day period (Fig 3). To avoid possible cross-contamination LAMP assays were performed into two batches of 14 samples each. When testing stool samples from infected rats with 40 iL3 (group 1) we obtained LAMP positive results continuously from day 6 p.i. -when parasite eggs were detected in feces for the first time- until the end of infection at day 28 (Fig 3A). When testing stool samples from infected rats with 400 iL3 (group 2) and 4,000 iL3 (group 3) we obtained in both groups LAMP positive results continuously from day 5 p.i. -one day before the onset of parasite eggs in feces- until the end of infection at day 28 (Fig 3B and 3C). Negative controls (pooled DNA samples from feces from non-infected rats; group 4) were never amplified and in all LAMP positive reactions a green fluorescence was clearly visualized under natural light.

### Examination of rat urine samples by LAMP

**Fig 4:**
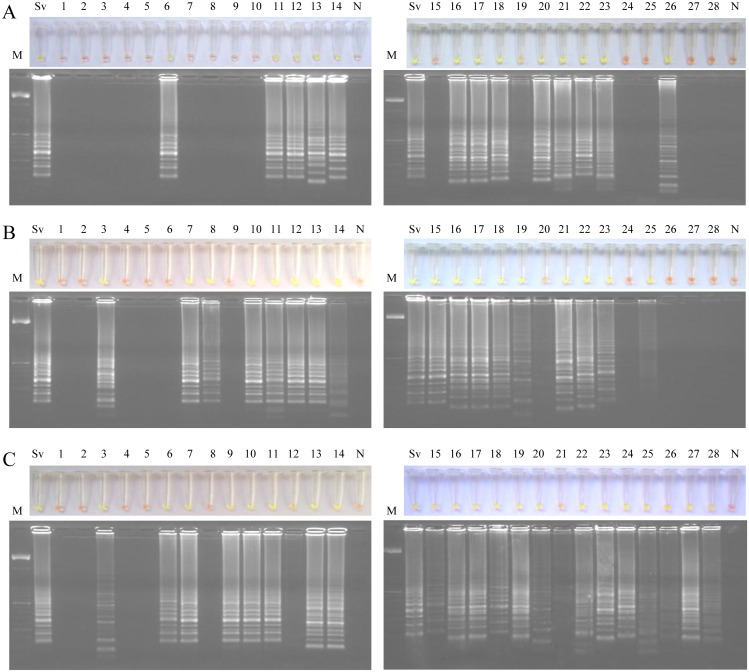
Examination of rats’ urine samples by Strong-LAMP assay. Analysis of each daily pool of urine samples obtained from each infection group of rats during a 28-day period. (A) Group 1, infected with 40 iL3. (B) Group 2, infected with 400 iL3. (C) Group 3, infected with 4,000 iL3. Lanes M, 50 bp DNA ladder (Molecular weight marker XIII, Roche); Lanes Sv, genomic DNA from *S*. *venezuelensis* (1 ng); lanes 1–28, pooled urine samples obtained at days 1–28 post-infection; lanes N, negative controls (pooled feces from non-infected rats, group 4).

LAMP assay was also performed in each daily pool of urine samples obtained from each infection group of animals during a 28-day period (Fig 4). The 28 urine samples obtained from each infection group were tested in two batches of 14 samples each. Analyses of urine samples from rats infected with 40 iL3 (group 1) showed LAMP positive results on days 6, 11–14, 16–23 and 26 p.i. (Fig 4A). Analyses of urine samples from rats infected with 400 iL3 (group 2) showed LAMP positive results on days 3, 7–8, 10–23 and 25 p.i. (Fig 4B). Finally, analyses of urine samples from rats infected with 4,000 iL3 (group 3) showed LAMP positive results on days 3, 6–7 and continuously from day 9 until the end of infection on day 28 (Fig 4C). Four urine samples considered as positive results (including those obtained on day 19 from group 1, on day 20 from group 2 and on days 12 and 21 from group 3) did not show a color change to green fluorescent as appreciable as other LAMP positive results, but a faint ladder-like pattern could be observed on agarose gel electrophoresis. We obtained DNA amplification in pooled urine sample from group 1 on the 6th day (the same day that parasite eggs were detected in feces for the first time), but regarding group 2 and group 3, we detected DNA amplification in pooled urine sample on the 3rd day (two days before than onset of parasite eggs in feces). Curiously, LAMP positive results were not obtained on those days that the maximal fecal egg count was observed in each infection group (i.e., day 10 for group 1, day 9 for group 2 and day 8 for group 3, respectively).

### Evaluation of LAMP on diagnosis of human strongyloidiasis

All patients´ stool samples were tested by RT-PCR and LAMP to compare results. The RT-PCR resulted positive in 6/7 patients´ stool samples with confirmed strongyloidiasis by parasitological tests previously applied. In addition, a positive result was obtained in a sample to which none parasitological test could be performed; negative results were obtained in negative parasitological samples for *S*. *stercoralis* (Table 1). All patients´ stool samples with confirmed strongyloidiasis by parasitological tests could be detected by LAMP, including the sample which resulted negative in previous RT-PCR analyses. In addition, as for RT-PCR, we also obtained a positive result in the sample to which parasitological tests were not available. Negative parasitological patients´ stool samples for strongyloidiasis resulted in a negative LAMP amplification, including those positive samples for *Taenia saginata* and "hookworn", respectively (Table 1; S3 Fig).

## Discussion

There are many difficulties in correctly diagnosing strongyloidiasis because most patients are asymptomatic and the lack of sensitivity and specificity of the commonly used parasitological and serological diagnostic methods, respectively [43]. Several PCR-based molecular methods offering high sensitivity and specificity have been recently proposed in diagnosing strongyloidiasis [25, 22, 26, 27]. A LAMP method could be an economic, simple and applicable alternative to PCR-based methods in field conditions for diagnostic assays [44]. On the other hand, all new PCR-based approaches for *Strongyloides* spp. DNA detection have been always mainly assayed for stool samples from both experimentally infected animals and clinical stool samples [45, 46, 22, 25, 26, 27] but no other biological samples, such as urine, have been investigated for molecular diagnostic purposes. In our work, we used a *S*. *venezuelensis* rodent model in order to test a new LAMP assay for diagnosing strongyloidiasis both in stool and, for the first time, urine samples. We used an experimental infection with *S*. *venezuelensis* since this parasitic nematode has been widely used as a tool and laboratory model for human and animal strongyloidiasis research [47, 48]. The use of a *S*. *venezuelensis* rodent model allowed us to collect well-defined stool and urine samples that would otherwise have been very difficult to obtain from human patients, including samples from recently acquired infections and samples with low parasite load resembling to those likely obtained in chronic human infections. Additionally, a classical parasitological diagnostic method, such as direct faecal examination by counting EPG was used for monitoring infection as well as to compare results in parallel with molecular assays.

Results obtained by counting EPG showed a similar dynamics of *S*. *venezuelensis* infection to that previously reported not only by this parasite or by *S*. *ratti* in Wistar rats [49] but also in Lewis rats [46] and in male Sprague-Dawley rats [50].

To design specific primers for our LAMP assay, a 329 nucleotide bp from the 5´ end of a linear DNA partial sequence in the 18S ribosomal RNA gene from *S*. *venezuelensis* was selected [39]. For *Strongyloides* species, 18S ribosomal RNA gene (rDNA) has been analyzed [51, 52, 39, 53]. It is considered that small subunit ribosomal RNA (SSU rDNA) sequences within *Strongyloides* species are all very similar making the resolution of their phylogeny problematic as many branch lengths are inferred to be very short when distance and likelihood methods are applied [39, 53]. Closer analysis of the SSU rDNA sequences from a number of *Strongyloides* species have been shown to identify a putative molecular synapomorphy (comprising 8 to 10 nucleotides) within the E9-2 stem-loop of the V2 variable region, thus allowing to distinguish two clades within *Strongyloides* genus: one containing *Strongyloides* spp. ex snake, *S*. *stercoralis* and *S*. *fuelleborni* (namely *"stercoralis"* clade, with a 10 nucleotides sequence: ATTTTATATT), and another containing *S*. *ratti*, *S*. *suis*, *S*. *venezuelensis*, *S*. *cebus*, *S*. *fuelleborni kelleyi* and *S*. *papillosus* (namely *"cebus"* clade, with a 8 nucleotides sequence: ATT—TTTTC) [39]. Among the set of primers automatically generated when designing LAMP for specific amplification of *S*. *venezuelensis*, the F2 primer was finally manually selected to be used since its sequence at 3´ end -which location serve as the replication starting point after annealing- would allow not only a specific annealing in the 8 nuleotides sequence of *"cebus"* clade but also, theoretically, in the 10 nucleotides sequence of *"stercoralis"* clade if present in samples. Thus, the LAMP assay may be employed for simultaneous detection of several *Strongyloides* species. At present, only *S*. *stercoralis* and *S*. *fuelleborni* are known to cause infection in humans but infection with other species might be possible. Besides, the designed LAMP can also be use in the *S*. *venezuelensis* experimental infection rodent model.

After verifying the operation and specificity of PCR F3-B3, we attempted to establish the most suitable reaction mixture for the set of primers operation in the LAMP assay. The limit of detection of the LAMP assay resulted tenfold higher when *Bst* polymerase 2.0 WarmStart was used in comparison with *Bst* polymerase 2.0 (corresponding to 0.01 ng *vs*. 0.1 ng, respectively). It has been previously reported a number of advantages of *Bst* polymerase WarmStart version compared to other commercially available versions, such as faster amplification [54], increased stability at room temperature [55] and also greater sensitivity [56, 57]. We emphasize the importance of setting up the best conditions and molecular components for primers set operation in a LAMP assay. When analyzing the stool samples, the Strong-LAMP resulted more sensitive than microscopy, at least in moderate and high levels of infection. A similar result has been also reported for RT-PCR in comparison to microscopy in detecting first-stage larvae of *S*. *ratti* in a rodent model infected subcutaneously with 2,500 iL3 [58]. When analyzing the urine samples daily collected, we obtained Strong-LAMP positive results during the course of infection depending on infection dose.

In a work carried out by Marra et al. [46], in which the migration route of *S*. *venezuelensis* was evaluated by PCR and histological analysis in Lewis rats infected subcutaneously with 4,000 iL3, it was noted that the appearance of larvae in alveoli was already clear at 48 h p.i.. It was also observed that at 72 h p.i. all infected animals had larvae in the lungs and no larvae were found in any other organs that were examined. It was at this time, at 72 h p.i., when we obtained Strong-LAMP positive results in urine samples from groups 2 and 3, thus indicating the possibility of detecting *S*. *venezuelensis* free circulating DNA as a consequence of destroying larvae passing through the lungs and ending up in urine. Also according to that study, at 120 h p.i. larvae begin to disappear from the lungs and were found inserted in the small intestine villosities at 48–72 h later. Interestingly, it is also at this time in our study (approximately on the 9th day p.i.) when urine samples from group 3 (infected with 4,000 iL3) resulted Strong-LAMP positive every day until the end of infection.

In group 2 (400 iL3) a first positive result was also obtained by Strong-LAMP in the pool of urine samples at 72 h p.i.; however, a time lag in the appearance of positive results until the end of infection in comparison to group 3 (4,000 iL3) was detected, possibly related to the lower initial infective dose. Such time lag in the appearance of positive results was much more apparent when testing urine samples from group 1; since group 1 was infected with the lowest infective dose of larvae (40 iL3), a first positive result obtained on day 6 p.i. would suggest that parasites reached the lungs later and consequently it would take longer to settle them in the small intestine villosities.

Unexpectedly, we did not obtained Strong-LAMP positive results in urine samples on days in which the maximal fecal egg count was observed in each infection group. The absence of information on this event or similar in already published data does not allow us to compare our results. We can only speculate on the possibility of some features related to the dynamics of the biological cycle of the parasite.

The potential clinical applicability of the Strong-LAMP could be demonstrated on a number of human clinical stool samples. We obtained positive results in those stool samples with both parasitological demonstration and confirmed detection by RT-PCR of *S*. *stercoralis*. In addition, the analysis of one sample (no. 496) with no parasitological test applied but positive by RT-PCR, resulted positive by Strong-LAMP. Moreover, another sample (no. 069) with parasitological demonstration of *S*. *stercoralis* but negative by RT-PCR resulted also positive by Strong-LAMP; however, considering the limited number of human samples tested it is difficult at this time to suggest a potential greater sensitivity of LAMP assay than RT-PCR in detecting *S*. *stercoralis* DNA in stool samples. Furthermore, confirmed negative stool samples for *S*. *stercoralis* both by parasitological and RT-PCR methods resulted negative by Strong-LAMP as well, even those samples infected with *T*. *saginata* and hookworm, thus corroborating once again the specificity of our designed LAMP assay for exclusively detection of *Strongyloides* spp. DNA. Although specificity was also previously determined *in silico* by using a thoroughly BLASTN search and alignment analysis in online databases and no cross-reaction of other sequences reported for possible human pathogens were detected, it is important to note that, considering the absence of a single gold standard for strongyloides diagnosis, and because LAMP products cannot be routinely sequenced to confirm identity, other micro-organisms that may be commonly found in stool samples (e.g. bacteria and fungi, such as *Candida* spp.) should be investigated in order to further validate the LAMP assay for human diagnosis.

In this work, we report for the first time, on the development of a new LAMP assay (Strong-LAMP) for sensitive detection of *S*. *venezuelensis* DNA in both stool and urine samples in a well-established Wistar rats experimental infection model. In addition, this Strong-LAMP assay can be also applied effectively for the detection of *S*. *stercoralis* DNA in patients´ stool samples. Clearly, in terms of potential human diagnostic, this assay requires additional validation using a greater number of clinical stool samples.

The successfully amplification of *Strongyloides* spp. DNA in infected urine samples by LAMP assay as well as the advantages that urine would have in collection, storage and processing in comparison to patients´ stool samples, should make us consider the possibility of starting to use urine specimens in diagnosing human strongyloidiasis.

However, it will be convenient to further consider the difference between the samples from a *S*. *venezuelensis* rodent model in acute disease and chronic human *S*. *stercoralis* infections. Since urine is actually an unusual requested biological sample from patients to detect *S*. *stercoralis*, further studies using clinical urine samples for human diagnostics of strongyloidiasis are strongly-(LAMP) recommended.

## Supporting Information

S1 Checklist Checkmarks for the STARD Checklist. (PDF)

S1 Flow Diagram A diagram showing experimental design and results. (PDF)

S1 Fig PCR verification, detection limit and specificity using outer primers F3 and B3. (A) PCR verification of expected 215 bp target length amplicon. Lane M, 50 bp DNA ladder (Molecular weight marker XIII, Roche); lanes Sv, *S*.*venezuelensis* DNA (1 ng); lanes N1 and N2, negative controls (no DNA template). (B) Detection limit of PCR. Lane M, 50 bp DNA ladder (Molecular weight marker XIII, Roche); lane Sv: *S*. *venezuelensis* DNA (1 ng); lanes 10^−1^–10^−6^: 10-fold serially dilutions of *S*. *venezuelensis* DNA; lane N, negative control (no DNA template). (C) Specificity of PCR. Lane M, 50 bp DNA ladder (Molecular weight marker XIII, Roche); lanes Sv, Sm, Sh, Sj, Si, Sb, Fh, Dd, Hd, Ec, Cd, Ll, Bp, As, Tt, Eg, Ts, Pm, Po, Pv, Eh, Gi, S. venezuelensis, S. mansoni, *S*. *mansoni*, *S*. *haematobium*, *S*. *japonicum*, *S*. *intercalatum*, *S*. *bovis*, *Fasciola hepatica*, *Dicrocoelium dendriticum*, *Hymenolepis diminuta*, *Equinostoma caproni*, *Calicophoron daubneyi*, *Loa loa*, *Brugia pahangi*, *Anisakis simplex*, *Taenia taeniformis*, *Echinococcus granulosus*, *Trichinella spiralis*, *Plasmodium malariae*, *P*. *ovale*, *P*. *vivax*, *Entamoeba histolytica*, *Giardia intestinalis* DNA samples (1 ng/each), respectively; lane N, negative control (no DNA template).
(TIF)

S2 Fig Setting up LAMP assay. (A) LAMP amplification results obtained using different polymerases tested in a heating block by the addition of SYBR Green I (up) or by visualization on agarose gel (down). Lane M, 50 bp DNA ladder (Molecular weight marker XIII, Roche); lanes FL, 2.0, WS, *Bst* polymerase Large Fragment, *Bst* polymerase 2.0, *Bst* polymerase WarmStart, respectively. Lane N: negative control (no DNA template). (B) Sensitivity assessment of LAMP assay performed with *Bst* polymerase 2.0. (C) Sensitivity assesment of LAMP assay performed with *Bst* polymerase WarmStart. For (B) and (C): lane M, 50 bp DNA ladder (Molecular weight marker XIII, Roche); lanes Sv, genomic DNA from *S*. *venezuelensis* (1 ng); lanes 10^−1^–10^−10^: 10-fold serially dilutions; lane N: negative controls (no DNA template). (D) Specificity of the LAMP assay for *S*. *venezuelensis*. A ladder of multiple bands of different sizes could be only observed in *S*. *venezuelensis* DNA sample. Lane M, 50 bp DNA ladder (Molecular weight marker XIII, Roche); lanes Sv, Sm, Sh, Sj, Si, Sb, Fh, Dd, Hd, Ec, Cd, Ll, Bp, As, Tt, Eg, Ts, Pm, Po, Pv, Eh, Gi, *S*. *venezuelensis*, *S*. *mansoni*, *S*. *mansoni*, *S*. *haematobium*, *S*. *japonicum*, *S*. *intercalatum*, *S*. *bovis*, *Fasciola hepatica*, *Dicrocoelium dendriticum*, *Hymenolepis diminuta*, *Equinostoma caproni*, *Calicophoron daubneyi*, *Loa loa*, *Brugia pahangi*, *Anisakis simplex*, *Taenia taeniformis*, *Echinococcus granulosus*, *Trichinella spiralis*, *Plasmodium malariae*, *P*. *ovale*, *P*. *vivax*, *Entamoeba histolytica*, *Giardia intestinalis* DNA samples (1 ng/each), respectively; lane N, negative control (no DNA template).
(TIF)

S3 Fig Examination of patients´ stool samples by Strong-LAMP. Analysis of patients´ stool samples included in the study by Strong-LAMP. In all positive LAMP results a green fluorescence was clearly visualized under natural light (up) or by electrophoresis in agarose gel (down). Lane M, 50 bp DNA ladder (Molecular weight marker XIII, Roche); lane Sv, genomic DNA from *S*. *venezuelensis* (1 ng); numbers 030, 140, 231, 232, 338, 339, 496, 069, 259, 331, 468, 126, stool samples from twelve patients; lanes N, negative controls (ultrapure water instead DNA as template).
(TIF)
