# Supplementary figures and images for: Strong-LAMP: A LAMP Assay for Strongyloides spp. Detection in Stool and Urine Samples. Towards the Diagnosis of Human Strongyloidiasis Starting from a Rodent Model
Source: PLoS Negl Trop Dis. 2016 Jul 14;10(7):e0004836. doi: 10.1371/journal.pntd.0004836 (PMC4945066; doi:10.1371/journal.pntd.0004836)

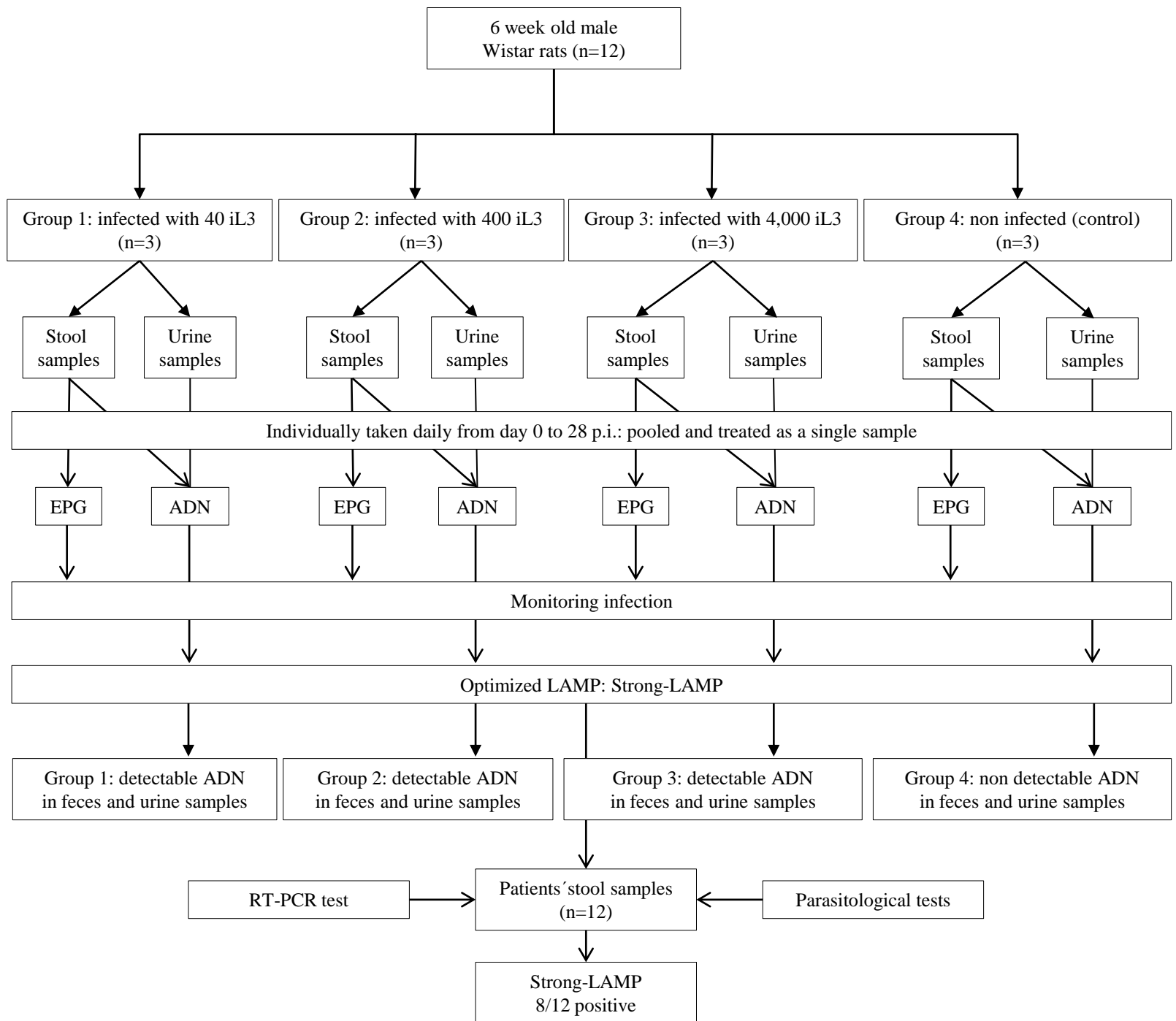

Supplement: S1 Flow Diagram — (PDF) [file pntd.0004836.s002.pdf]

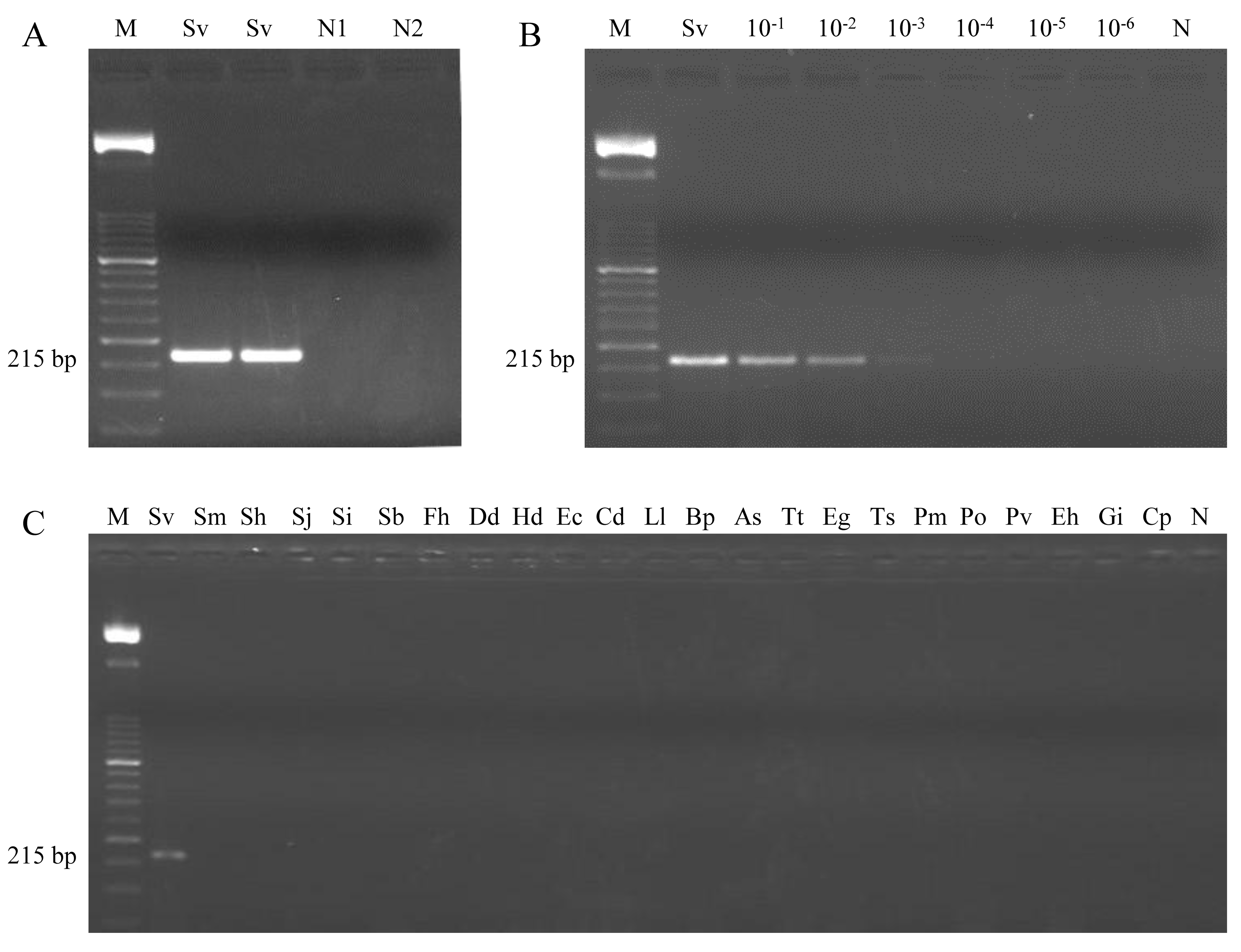

Supplement: S1 Fig — (A) PCR verification of expected 215 bp target length amplicon. Lane M, 50 bp DNA ladder (Molecular weight marker XIII, Roche); lanes Sv, S.venezuelensis DNA (1 ng); lanes N1 and N2, negative controls (no DNA template). (B) Detection limit of PCR. Lane M, 50 bp DNA ladder (Molecular weight marker XIII, Roche); lane Sv: S. venezuelensis DNA (1 ng); lanes 10−1–10−6: 10-fold serially dilutions of S. venezuelensis DNA; lane N, negative control (no DNA template). (C) Specificity of PCR. Lane M, 50 bp DNA ladder (Molecular weight marker XIII, Roche); lanes Sv, Sm, Sh, Sj, Si, Sb, Fh, Dd, Hd, Ec, Cd, Ll, Bp, As, Tt, Eg, Ts, Pm, Po, Pv, Eh, Gi, S. venezuelensis, S. mansoni, S. mansoni, S. haematobium, S. japonicum, S. intercalatum, S. bovis, Fasciola hepatica, Dicrocoelium dendriticum, Hymenolepis diminuta, Equinostoma caproni, Calicophoron daubneyi, Loa loa, Brugia pahangi, Anisakis simplex, Taenia taeniformis, Echinococcus granulosus, Trichinella spiralis, Plasmodium malariae, P. ovale, P. vivax, Entamoeba histolytica, Giardia intestinalis DNA samples (1 ng/each), respectively; lane N, negative control (no DNA template). (TIF) [file pntd.0004836.s003.tif]

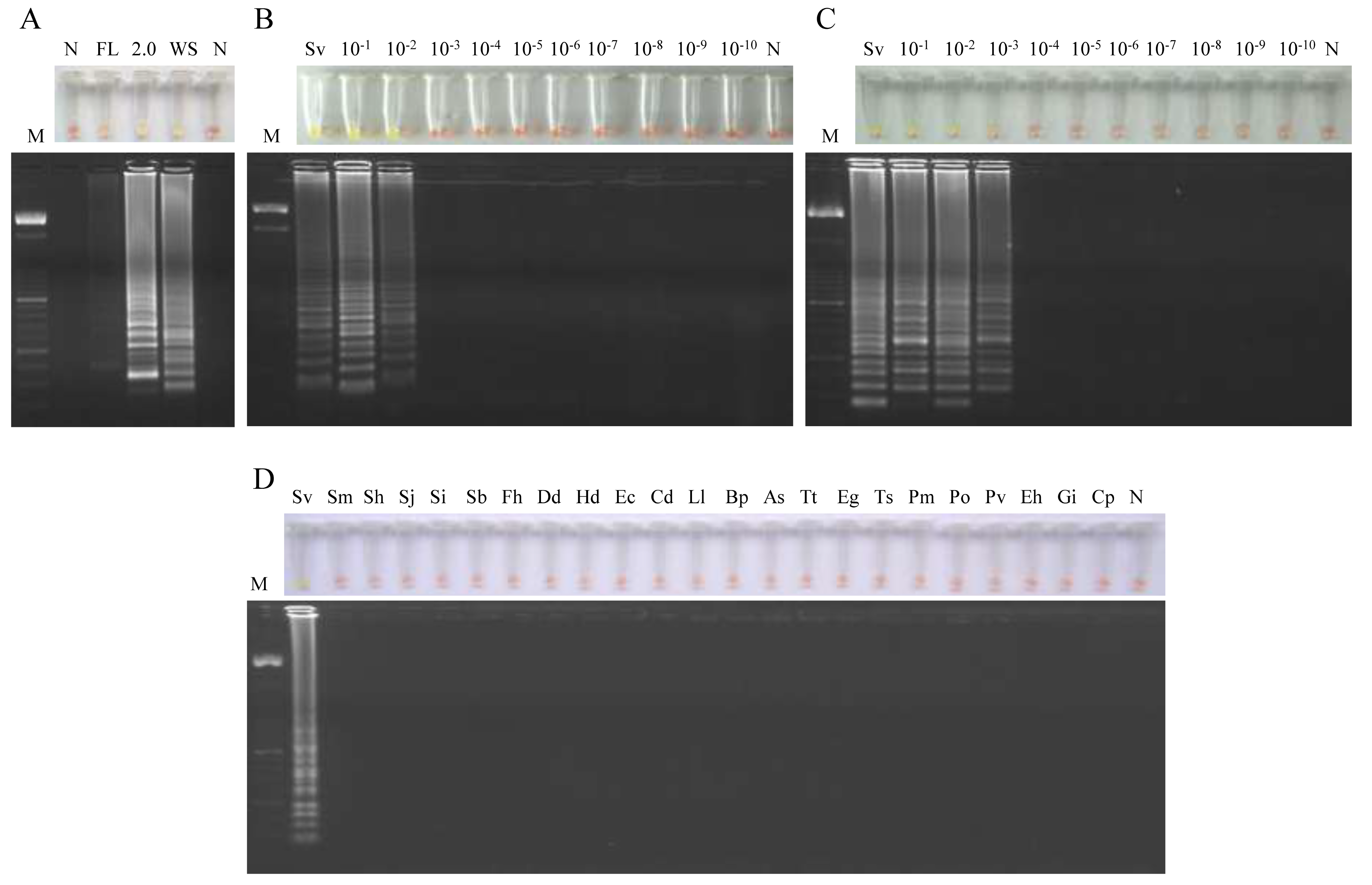

Supplement: S2 Fig — (A) LAMP amplification results obtained using different polymerases tested in a heating block by the addition of SYBR Green I (up) or by visualization on agarose gel (down). Lane M, 50 bp DNA ladder (Molecular weight marker XIII, Roche); lanes FL, 2.0, WS, Bst polymerase Large Fragment, Bst polymerase 2.0, Bst polymerase WarmStart, respectively. Lane N: negative control (no DNA template). (B) Sensitivity assessment of LAMP assay performed with Bst polymerase 2.0. (C) Sensitivity assesment of LAMP assay performed with Bst polymerase WarmStart. For (B) and (C): lane M, 50 bp DNA ladder (Molecular weight marker XIII, Roche); lanes Sv, genomic DNA from S. venezuelensis (1 ng); lanes 10−1–10−10: 10-fold serially dilutions; lane N: negative controls (no DNA template). (D) Specificity of the LAMP assay for S. venezuelensis. A ladder of multiple bands of different sizes could be only observed in S. venezuelensis DNA sample. Lane M, 50 bp DNA ladder (Molecular weight marker XIII, Roche); lanes Sv, Sm, Sh, Sj, Si, Sb, Fh, Dd, Hd, Ec, Cd, Ll, Bp, As, Tt, Eg, Ts, Pm, Po, Pv, Eh, Gi, S. venezuelensis, S. mansoni, S. mansoni, S. haematobium, S. japonicum, S. intercalatum, S. bovis, Fasciola hepatica, Dicrocoelium dendriticum, Hymenolepis diminuta, Equinostoma caproni, Calicophoron daubneyi, Loa loa, Brugia pahangi, Anisakis simplex, Taenia taeniformis, Echinococcus granulosus, Trichinella spiralis, Plasmodium malariae, P. ovale, P. vivax, Entamoeba histolytica, Giardia intestinalis DNA samples (1 ng/each), respectively; lane N, negative control (no DNA template). (TIF) [file pntd.0004836.s004.tif]

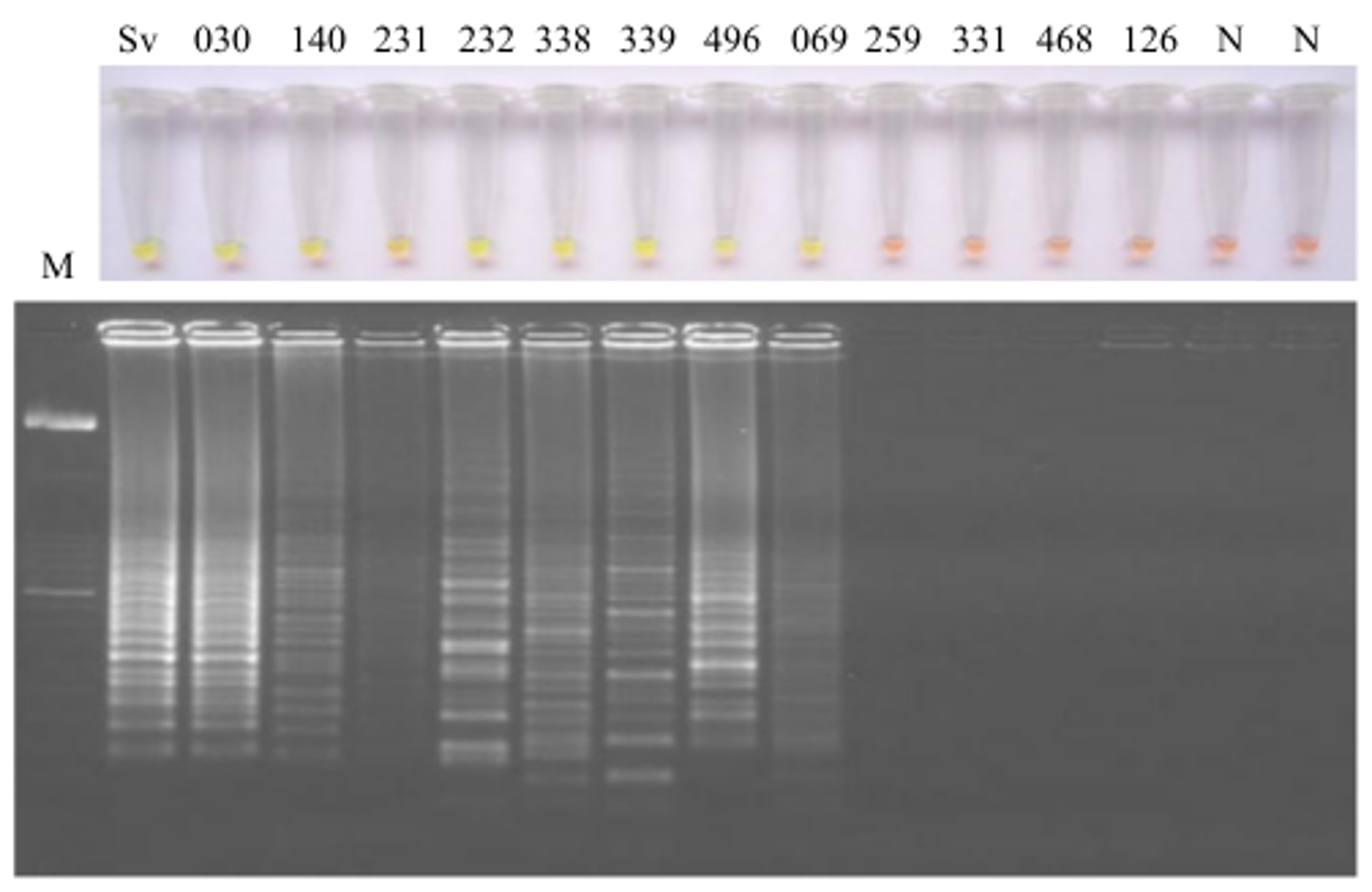

Supplement: S3 Fig — Analysis of patients´ stool samples included in the study by Strong-LAMP. In all positive LAMP results a green fluorescence was clearly visualized under natural light (up) or by electrophoresis in agarose gel (down). Lane M, 50 bp DNA ladder (Molecular weight marker XIII, Roche); lane Sv, genomic DNA from S. venezuelensis (1 ng); numbers 030, 140, 231, 232, 338, 339, 496, 069, 259, 331, 468, 126, stool samples from twelve patients; lanes N, negative controls (ultrapure water instead DNA as template). (TIF) [file pntd.0004836.s005.tif]
